# Long-term fenofibrate treatment impaired glucose-stimulated insulin secretion and up-regulated pancreatic NF-kappa B and iNOS expression in monosodium glutamate-induced obese rats: Is that a latent disadvantage?

**DOI:** 10.1186/1479-5876-9-176

**Published:** 2011-10-14

**Authors:** Shuai-nan Liu, Quan Liu, Lin-yi Li, Yi Huan, Su-juan Sun, Zhu-fang Shen

**Affiliations:** 1 State Key Laboratory of Bioactive Substances and Functions of Natural Medicines, Institute of Materia Medica, Chinese Academy of Medical Sciences and Peking Union Medical College, No.1 Xiannongtan Street, Beijing 100050, P. R. China

## Abstract

**Background:**

Fenofibrate, a PPAR alpha agonist, has been widely used in clinics as lipid-regulating agent. PPAR alpha is known to be expressed in many organs including pancreatic beta cells and regulate genes involved in fatty acid metabolism. Some reports based on cell lines or animals have provided evidences that PPAR alpha agonists may affect (increased or suppressed) beta cell insulin secretion, and several studies are producing interesting but still debated results.

**Methods:**

In this research, we investigated the long term effects of fenofibrate on beta cell function in a metabolic syndrome animal model, monosodium glutamate (MSG) induced obese rats. Obese MSG rats were administered by gavage with fenofibrate at a dose of 100 mg/kg for 12 weeks. Oral glucose tolerance and insulin tolerance tests were performed to evaluate glucose metabolism and insulin sensitivity. We have used the hyperglycemic clamp technique to evaluate the capacity of beta cell insulin secretion. This technique provides an unbiased approach to understand the beta cell function in vivo. The changes of gene and protein expression in the pancreas and islets were also analyzed by Real-Time-PCR, Western blot and immunostaining.

**Results:**

Fenofibrate reduced the plasma lipid levels within a few days, and showed no beneficial effects on glucose homeostasis or insulin sensitivity in obese MSG rats. But the animals treated with fenofibrate exhibited significantly decreased fasting plasma insulin and impaired insulin secretory response to glucose stimulation. Further studies confirmed that fenofibrate increased MDA level and decreased total ATPase activity in pancreatic mitochondrion, accompanied by the upregulation of iNOS and NF-kappa B and TNF alpha expression in pancreatic islets of obese MSG rats.

**Conclusions:**

Long-term fenofibrate treatment disrupted beta cell function, and impaired glucose-stimulated insulin secretion in obese MSG rats, perhaps to some extent associated with the activated inflammatory pathway and increased formation of oxidative products, especially the up-regulation of NF-kappa B and iNOS expression in islets.

## Background

Peroxisome proliferator-activated receptor alpha is an important mediator of fatty acids metabolism by regulating the expression of genes involved in lipid metabolism [1]. Many reports suggested that PPAR alpha activation could improve the peripheral insulin resistance by relieving lipid-mediated inhibition of insulin-stimulated glucose disposal in both rodents and humans [2,3]. Furthermore, PPAR alpha is also known to be expressed in islets. Many studies have indicated that some PPAR alpha agonists (the class of Fibrates) may affect insulin secretion *in vitro *[4]. Some evidences showed that PPAR alpha activation improved insulin secretion at low glucose concentrations in isolated rat pancreatic islets [5-9]. While PPAR alpha overexpression also stimulated fatty acid oxidation and impaired beta cell function independent of triglyceride content in insulinoma cells [10]. In some extent, such results of PPAR alpha agonist on insulin secretion showed a certain contradictory.

In order to confirm the direct effect of a PPAR alpha agonist, fenofibrate, on beta cell function *in vivo*, we chose obese MSG rats to identify insulin secretion after long-term fenofibrate treatment. The monosodium glutamate (MSG) neonatal administration after birth in mice or rats provides a model of obesity, which have been used as an metabolic dysfunction animal model and exhibited impaired glucose tolerance and insulin resistance for about 4-6 months old [11,12].

Our primary work has shown that fenofibrate treatment significantly decreased fasting plasma insulin in obese MSG rats without improving insulin sensitivity, we hypothesize that fenofibrate may cause an adverse effect on insulin secretion in this model.

Recently, it was emphasized that PPAR alpha has both direct effects on the islet itself and indirect effects via modulation of the mediators involved in insulin secretion and systemic inflammatory. Besides, beta cells were known to be particularly susceptible to reactive oxygen species damage since they have relatively low expression of antioxidant enzymes [13-15]. The augmented reactive oxygen or nitrogen species (ROS/RNS) generation may play an important role in the diminished activity of beta cells. Therefore, we observed the circulating and pancreatic SOD, MDA, NO content to confirm the effect of fenofibrate on ROS/RNS. The metabolic factors invovled in insulin secretion process, including intracellular ion transport, mitochondrial ATP production, are discussed. ATPase is involved in maintaining ions gradients across the beta cell plasma membrane, and is thought to modulate the ATP level for the maintenance of homeostasis [16,17]. In addition, several pancreatic protein and enzymes, which were involved in insulin biosynthesis, exocytosis, energy metabolism and inflammatory pathway, were also associated with beta cells insulin secretory function.

## Methods

### Animals

Wistar rats were purchased from the Institute of Laboratory Animal Science, CAMS & PUMC. Monosodium glutamate (MSG) at a dose of 4 g/kg body weight was subcutaneously injected to neonatal Wistar rats once daily for 7 consecutive days after birth to induce obese rat model. Compared to the normal rats, fasting plasma concentrations of triglyceride and insulin were significantly elevated in obese MSG rats at the age of about six months. At this time, the animals exhibited obvious features of insulin resistance and visceral obesity, and could be considered as a typical metabolic syndrome animal model. There are three experiment groups in this study: Normal rats group (Nor), obese MSG rats control group (Con), obese MSG rats treated with fenofibrate group (Fen). 6 months old obese MSG rats with insulin resistance (Male, average body weight 775 g) were selected and divided into two groups (n = 12/group) as Con and Fen: Con group was orally administered by gavage with water and Fen group with fenofibrate (Beijing Yimin pharmaceutical Co. 100 mg fenofibrate/tablet) at a dose of 100 mg/kg per day for about 12 weeks. Ten fenofibrate tablets were ground and dissolved into 50 ml water to prepare suspension for gavaging 0.5 ml/100 g body weight every day. Age matched normal rats (Male, average body weight 529 g, n = 12) were used as Nor group treated with water. Throughout the 12-weeks administration period, body weight, water and food intake were recorded weekly. Four hours fasting glycaemia and lipid levels were measured fortnightly and plasma insulin concentrations were measured at days 18, 40 and in clamp test. Oral glucose tolerance tests (OGTTs) were conducted on day 14, 28 and 41, insulin tolerance tests (ITT) on day 25 and 56. After 12 weeks treatment, six animals from each group were evaluated the beta cell function by hyperglycemic clamp. The remaining animals were euthanized for plasma biochemical indices assay and the pancreas was excised for determination of some pancreatic specific mRNA and protein levels. All animals were housed in an air-conditioned room with a constant 12 h light/dark cycle at 23 ± 2°C., and had free access to water and standard chow. All animals were handled in accordance with The Standards for Laboratory Animals (GB14925-2001) and The Guideline on the Humane Treatment of Laboratory Animals (MOST 2006a) established by the People's Republic of China. The two guidelines were conducted in adherence to the regulations of Institutional Animal Care and Use Committee (IACUC) and all animal protocols were approved by IACUC.

### Oral glucose tolerance test (OGTT) and insulin tolerance test (ITT)

Rats were fasted for 4 h and blood was sampled from tail for glucose assay by the glucose oxidase method at baseline (as 0 min) and 30, 60 and 120 min after glucose (2 g/kg) loading in OGTT or 40 and 90 min after insulin (0.4 IU/kg) subcutaneous injection in ITT.

### Hyperglycemic clamp

The clamp was performed on 12th week after treatment. On the tested day, the animals were fasted for 10 h and then anesthetized with pentobarbital sodium (50 mg/kg body weight, intraperitoneally) and placed on a heating pad at 37°C. The anesthetics were reinforced every 60 min. The right jugular vein was catheterized (Micro-renathane, 0.025 × 0.012") for the infusion of glucose and the right femoral artery for the sampling. After operation, the animal rested for 30 min to lessen the stimulation. Then D-glucose was rapidly injected at a dose of 0.25 g/kg body weight in 1 min, followed a continuous infusion of glucose (50%) at a rate of 10-20 μl/min [18,19]. Blood samples were collected initially (0 min) and then every 5 or 10 min throughout the test. Plasma glucose concentration was monitored instantaneously to adjust the infusion rate of glucose and keep a hyperglycemia nearly 14 ± 0.5 mmol/l. Blood samples of 100 μl were taken in heparinized tubes at 0, 5, 10 min and the plasma was stored at -20°C for insulin analysis.

### Preparation of pancreas and pancreatic mitochondria

Half of the animals were euthanized after twelve weeks-treatment. The pancreas was immediately excised, rinsed, blotted, and weighed. Then the pancreas was cut into four fractions. One fraction was fixed in 4% paraformaldehyde for immunofluorescence analysis and the left were stored at -80°C for further analysis. Pancreatic tissue samples (100 mg) were homogenized in ice-cold buffer (10 mM TRIS-HCl, pH7.5, 1 mM EDTA, 250 mM sucrose). After the centrifugation, the supernatant was collected for assessment of lipid level and inflammatory products in the pancreas.

### Biochemical analysis

Plasma glucose was measured by the glucose oxidase method, and plasma levels of triglycerides (TG), total cholesterol (TC), high density lipoprotein-cholesterol (HDL-C), low density lipoprotein-cholesterol (LDL-C), Malondialdehyde (MDA), superoxide dismutase (SOD), nitric oxide (NO) and pancreatic mitochondrial ATPase activity were determined by enzymatic colorimetric methods with commercial kits (BioSino Inc.). Plasma insulin was measured by radioimmunoassay kit (Beijing Northern Bio. Inc.). Protein content was assayed according to the method of Lowry, using bovine serum albumin (BSA) as standard [20].

### Real Time PCR

Total RNA was extracted from pancreatic tissues from each group (4-6 animals) using TRIzol (Invitrogen). Total RNA (2 μg) was used for reverse transcription (RT) to synthesize complementary DNA with M-MLV reverse transcriptase, and real-time fluorescent detection PCR analysis was performed using Sybr-Green Dye PCR Master Mix (Takara Biotechnology Co. Ltd.) according to ABI 7000 PCR instrument recommendations (Applied Biosystems Japan Ltd., Tokyo, Japan). The theoretical basis for quantitation using real-time PCR has been described elsewhere [21]. Quantitative Real-time PCR analysis was carried out with the following cycle profile: 1 cycle at 95°C for 30 s, and 40 cycles at 95°C for 5 s, 60°C for 31 s.

Primers sequences were as follows: *iNOS *5'-CTGAAGCACTAGCCAGGGAC-3' and 5'-CAAATGTGCTTGTCACCACC-3'; *NF-kappa B *5'-ACAGTAGAGAAGTTGTATGCAGC-3' and 5'-GTGAGGTAGGTATCTGAGGCA-3'; *PPARalpha *5'-AGAGCCCCATCTGTCCTCTC-3' and 5'-ACTGGTAGTCTGCAAAACCAAA-3'; *UCP2 *5'-ACTTTCCCTCTGGATACCGC-3' and 5'-ACGGAGGCAAAGCTCATCTG-3'.

### Immunofluorescence

Paraffin-embedded pancreas tissues were cut into 2 μm sections and dewaxed using xylene, rehydrated through serial dilutions of ethyl alcohol [22]. The sections were washed and incubated with the antibodies diluted in 150 mM NaCl, 0.05% Tween-20, and 10 mM Tris-HCL (pH7.4). Primary antibodies used were mouse anti-insulin and rabbit polyclonal NOS2 antibody (diluted 1:50; Santa Cruz Biotechnology). Secondary antibodies used were FITC-conjugated goat anti-rabbit IgG and TRITC-conjugated goat anti-mouse IgG (Beijing zhongshanjinqiao Co.). Imaging was performed using a Leica TCS SP2 laser scanning confocal microscope (Nikon), and images were analyzed by the Image pro plus software.

### Western blot

Tissue samples were homogenized in lysis buffer (50 mM TRIS-HCl, 2% SDS, 10% glycerol) supplemented with protease inhibitor cocktail (P1265, Applygen Inc.). The homogenate was centrifuged at 12000 g for 10 min and the supernatant was taken to determine the protein concentrations. The soluble proteins were quantitated following the Lowry method. Equal amount of samples were resolved electrophoretically on a 10% sodium dodecyl polyacrylamide gel and transferred to polyvinylidene difluoride membranes. The membranes were probed using standard procedures and the signal was visualized by using an enhanced chemiluminescence detection system (ChemiScope2850, CLiNX science Instruments). Protein band densities were analyzed using Gel-Pro-Analyzer 3.1 software.

### Statistical analysis

Data were expressed as the mean ± standard error of the mean (SEM). The data obtained in the present study were analyzed using an ANOVA. A p value < 0.05 was considered to be statistically significant.

## Results

### Fenofibrate showed no improvement in glucose homeostasis but decreased plasma insulin level in obese MSG rats

**Figure 1:**
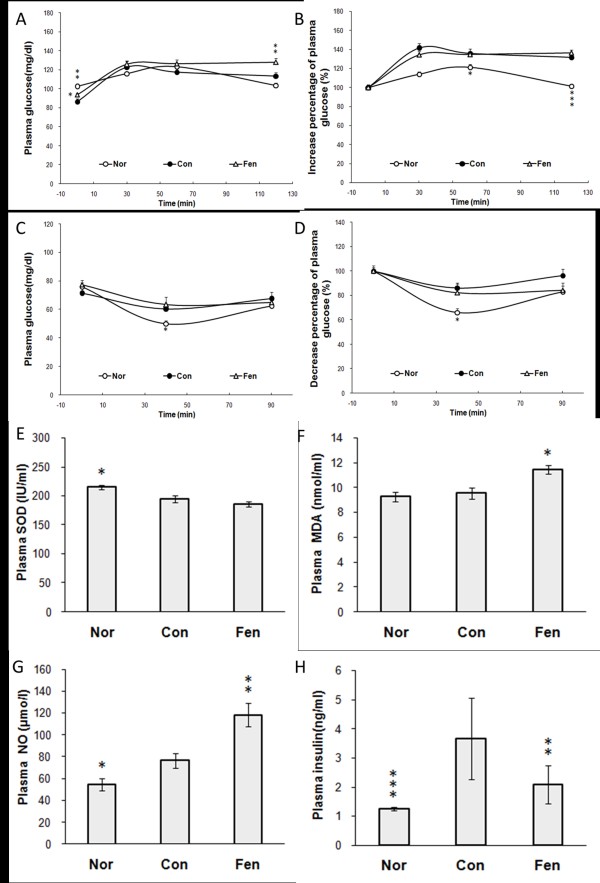
**Fenofibrate showed no improvement in glucose homeostasis but decreased plasma insulin level in obese MSG rats**. (A) Blood glucose level during the OGTT. (B) Increased percentage of blood glucose at each time point in OGTT. (C) Blood glucose level during the ITT. (D) Decreased percentage of glucose level at each time point in ITT. OGTT was performed in 4 h fasted animals on 6th week of treatment and ITT on 7th week. (E) Plasma superoxide dismutase level, (F) Plasma malondialdehyde level, (G) Plasma nitric oxide level, and (H) Plasma insulin level in obese MSG rats treated with water or fenofibrate. Nor, normal rats. Con, water treated obese MSG rats. Fen, fenofibrate treated obese MSG rats. Values represented means ± s.e.m. (n = 10-12). *p < 0.05, **p < 0.01, ***p < 0.001 vs. control.

The fasting blood glucose level was slightly higher (p <0.05) in fenofibrate treated group, compared to control group. Additionally, plasma glucose level at 120 min in OGTT was higher in fenofibrate treated group than control group (p <0.01, Figure 1A). However, AUC in both OGTT and ITT had no significant difference between fenofibrate treated group and control group (Figure 1B and 1D).

Some representative indices of anti-oxidative system were investigated during the administration. Plasma SOD level in obese MSG rats was lower than in normal group (194.4 ± 6.6 *versus *214.6 ± 4.2 IU/ml, p <0.05). Fenofibrate treatment failed to improve plasma SOD level (Figure 1E), and resulted in distinguished augmentation in plasma MDA content, a typical oxidative stress marker (11.4 ± 0.4 *versus *9.5 ± 0.5 nmol/ml, p <0.05, Figure 1F). Meanwhile, plasma NO was significantly 54.5 percent higher in fenofibrate group than in control group (118.2 ± 10.8 *versus *76.4 ± 7.1 μmol/l, p <0.01, Figure 1G). Fasting plasma insulin level was elevated in obese MSG rats with insulin resistance. Interestingly, although failed to improve insulin tolerance, fenofibrate treatment led to the decrease of plasma insulin level by 43.3% compared to control group (2.1 ± 0.7 *versus *3.7 ± 1.4 ng/ml, p <0.01, Figure 1H).

### Fenofibrate alleviates dyslipidemia in obese MSG rats

**Figure 2:**
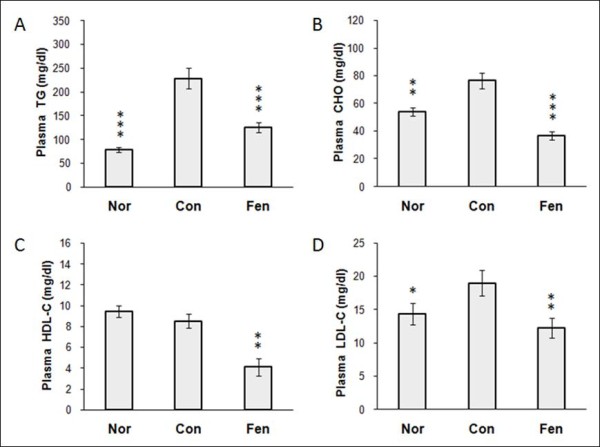
**Fenofibrate alleviates dyslipidemia in obese MSG rats**. Fasting cholesterol, triglyceride and major lipoproteins levels were assayed on 4th week. Nor, normal rats. Con, water treated obese MSG rats. Fen, fenofibrate treated obese MSG rats. Values represented means ± s.e.m. (n = 10-12). *p < 0.05, **p < 0.01, ***p < 0.001 vs. control.

Obese MSG rats developed hypercholesterolaemia and hypertriglyceridaemia with increased plasma TG and TC levels (Figure 2A and 2B), and higher plasma LDL-C fractions (Figure 2D) compared to normal group. Notably, fenofibrate treatment could alleviate the dyslipidemia, leading to 52% reduction in plasma cholesterol level and 45% reduction in triglyceride level, as well as reduced LDL-C and HDL-C levels (Figure 2).

### Fenofibrate affects insulin secretion in obese MSG rats

**Figure 3:**
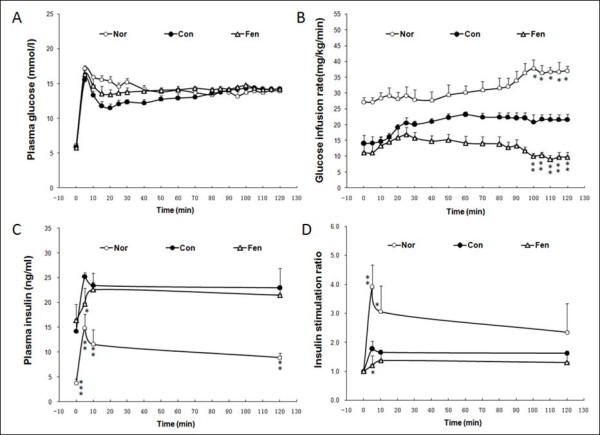
**Fenofibrate affects insulin secretion in obese MSG rats**. Pancreatic beta cell function was evaluated by hyperglycemic clamp. After sampling (t = 0 min) for the assay of the basal blood glucose and insulin, animals received intravenously a glucose bolus followed by a constant infusion of glucose to maintain plasma glucose level at 14 mmol/l. (A) Plasma glucose level, (B) Glucose infusion rates (GIR), (C) Plasma insulin level, and (D) Insulin stimulation ratio. Nor, normal rats. Con, water treated obese MSG rats. Fen, fenofibrate treated obese MSG rats. Values represented means ± s.e.m. (n = 6-8). *p < 0.05, **p < 0.01, ***p < 0.001 vs. control.

Next we performed hyperglycemic clamp experiments to evaluate beta cell function. A rapid rise in plasma glucose level appeared within 5 min in the three groups (Figure 3A). Thereafter, plasma glucose level was clamped at relatively constant level of 14-14.5 mmol/l for 120 min. The glucose infusion rate (GIR), an index of the maximum capacity of beta cell insulin secretion, was significantly 34 percent lower in obese MSG rats than in normal group (p <0.05). Fenofibrate treatment significantly decreased GIR by 52.7% compared to control group (10.2 ± 2.4 *vs*. 21.6 ± 3.5 mg/kg/min, Figure 3B). The dual phase of insulin secretion was observed in obese MSG rats and normal rats. The insulin level in model group was higher at each time point than in normal group (p <0.01). However, the increase ratio of plasma insulin at 5 min in obese MSG rats remained 54.7 percent lower than the normal group (p <0.01). The insulin peak at 5 min, namely first secretion phase, was almost absent after fenofibrate treatment compared to control group (Figure 3C and 3D, p <0.05).

### Fenofibrate increases MDA level and decreases ATPase activity in pancreatic mitochondrion in obese MSG rats

**Figure 4:**
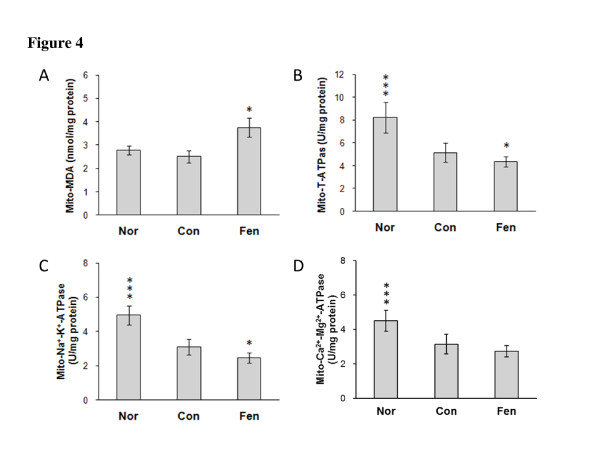
**Fenofibrate increases MDA level and decreases ATPase activity in pancreatic mitochondrion in obese MSG rats**. (A) MDA level, (B) Total ATPase Activity, (C) Na^+^-K^+^-ATPase activity and (D) Ca^2+^-Mg^2+^-ATPase activity. Nor, normal rats. Con, water treated obese MSG rats. Fen, fenofibrate treated obese MSG rats. Values represented means ± s.e.m. (n = 5-6). *p < 0.05, ***p < 0.001 vs. control.

Fenofibrate treatment caused a significant 49% increase of MDA level in pancreatic mitochondrion compared to control group (Con, 2.5 ± 0.7; Fen, 3.8 ± 1.0, p <0.05; Figure 4A). Pancreatic mitochondrial total ATPase activity in MSG obese rats was 37.4 percent lower than normal rats, and fenofibrate group showed a decreased activity of total ATPase by 15.5% than control group (Nor, 8.2 ± 1.3; Con, 5.2 ± 0.9; Fen, 4.4 ± 0.4. p <0.05, Figure 4B). Moreover, the activities of pancreatic mitochondrial Na^+^-K^+^-ATPase in fenofibrate-treated group were lower than that in control group (Figure 4C).

### Fenofibrate upregulates NF-κB and iNOS expression in the pancreas in obese MSG rats

**Figure 5:**
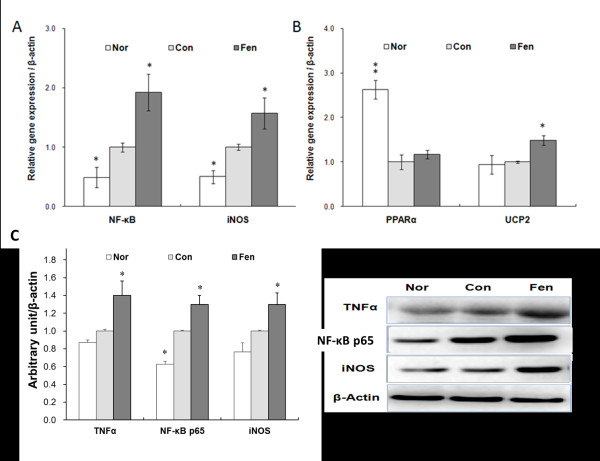
**Fenofibrate upregulates NF- kappaB and iNOS expression in the pancreas in obese MSG rats**. Quantitative RT-PCR analysis of pancreatic *NF- kappaB *and *iNOS *mRNA levels (A), *UCP2 *and *PPAR alpha *mRNA levels (B) in the three groups. A comparative threshold cycle (CT) method was used for relative quantification of gene expression using β-actin for normalization. Measurements were carried out in triplicate for each sample. (C) Western blot analysis of pancreatic NF- kappaB p65, iNOS and TNF alpha protein levels in three groups. Beta actin served as loading control. Data represented the mean of at least three independent experiments ± s.e.m. *p < 0.05, ** p < 0.01 vs. control.

To explore the molecular mechanism responsible for the biochemical changes induced by fenofibrate treatment, we examined mRNA level of several genes involved in insulin secretory process in the pancreas. Quantitative RT-PCR analysis showed that *NF- kappaB *and *iNOS *mRNA levels in the pancreas were increased by 1.9 fold and 1.6 fold in fenofibrate treated group compared to control group, respectively (p <0.01, Figure 5A). Uncoupling-protein 2 (*UCP2*), a mitochondrial protein involved in cellular oxidant defense and ATP generation, was found to be upregulated by 1.5 fold in fenofibrate treated group compared to control group (Figure 5B). *PPAR alpha *mRNA level in the pancreas was downregulated by 2.6 fold in obese MSG rats compared to normal group, but it was not significantly changed after fenofibrate treatment (Figure 5B).

**Figure 6:**
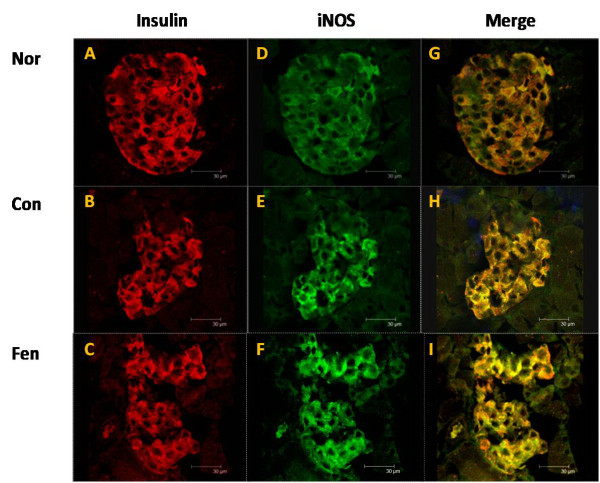
**Co-localization of iNOS and insulin in islets, fenofibrate upregulated iNOS expression in the islet beta cells**. Representative confocal microscopy images showed the co-localization of iNOS and insulin in islets. Paraffin-embedded pancreas sections were double immunostained by anti-insulin antibody and TRITC secondary (red) and anti-iNOS antibody and FITC secondary (green). A-C: insulin immunoreactivity; D-F: iNOS immunoreactivity; G-I: co-localization of insulin-iNOS indicated by yellowish fluorescence. Scale bar: 30 μm. Nor, normal rats. Con, water treated obese MSG rats. Fen, fenofibrate treated obese MSG rats.

The Western blot results showed that protein levels of iNOS, TNF alpha and NF- kappaB p65 domain were significantly higher in the pancreas after fenofibrate treatment (Figure 5C). Double immunolabeling of islets from fenofibrate treated animals showed that most iNOS immunoreactive cells also exhibited insulin immunoreactivity (Figure 6F). And the upregulation of iNOS expression in an islet in fenofibrate group accounted for 50-60 percent of total primary beta cells as shown in 6F section compared with that (30 percent upregulation) of control group in E section. Positive stained up-regulated iNOS expression islets covered around 70 percent of all analyzed islets in fenofibrate treated animals. These results demonstrate that fenofibrate upregulates iNOS expression in islet beta cells in obese MSG rats.

## Discussion

PPAR alpha controls several metabolic pathways of lipid and glucose metabolism. Fibrates as PPAR alpha agonists are clinically used to treat dyslipidemia in obesity, metabolic syndrome and type 2 diabetes individuals. PPAR alpha is expressed in pancreatic islets, and its agonist has been reported to improve pancreatic beta cell function in insulin-resistant rodents [23]. However, other studies showed that PPAR alpha agonists produced a decrease in glucose-stimulated insulin secretion in beta cells. Thus it remains unclear whether long term use of PPAR alpha agonist in pre-diabetic or diabetic animal models is benefit to beta cell function or not, although PPAR alpha has both direct and indirect effects on the islet including the modulation of systemic insulin sensitivity [24,25].

The initial objective of this study was to observe the effect of PPAR alpha agonist on lipid metabolism as well as insulin resistance in obese MSG rats. The effects of fenofibrate on lipid levels at the dose of 100 mg per day for 4 weeks were also assessed. Fenofibrate therapy lowered triglycerides and LDL-C level as well as HDL-C as shown in Figure 2D. These results showed some variance to that from clinical data in humans. Many researches have confirmed that in rodents fibrates may decrease HDL-C as a result of both decreased expression of HDL apolipoproteins, apoA-I and apoA-II and the metabolism enzymes such as hepatic lipase [26-28]. But in humans fibrates have a positive effect on HDL cholesterol as well as on apoA-I and apoA-II concentrations. This different regulatory effects seems to be caused by differences apoA-I gene promoter in the two species [29,30].

We found that the fasting plasma insulin level showed a notable reduction after fenofibrate treatment as shown in Figure 1H. And we have also found the similar results in the normal Wistar rats treated with fenofibrate for 4 weeks. It is well established that plasma insulin level is decreased due to improved insulin sensitivity in tissues (the liver and muscle, two major targets of insulin action) in obesity and Type 2 diabetes. However, our results showed that fenofibrate did not improve insulin sensitivity in obese MSG rats. Thus we wondered how plasma insulin level was decreased and speculated that insulin secretion may be impaired in obese MSG rats after fenofibrate treatment. To test our hypothesis, we measured insulin secretion *in vivo *with the hyperglycemic clamp which allowed for the quantitation of pancreatic insulin release in response to a defined hyperglycemic stimulus. The hyperglycemic clamp has been demonstrated to be a reliable technique to evaluate insulin secretory function. The results in Figure 3 demonstrated that fenofibrate treated obese MSG rats exhibited impaired insulin release in response to glucose stimulation. Taken together, these data suggest that decreased plasma insulin level in fenofibrate treated obese MSG rats is not an adaptive response to insulin sensitivity, but is a direct consequence of reduced insulin secretion in pancreatic beta cells.

While the mechanism by which fenofibrate impairs insulin secretion is not completely understood, numerous studies have shown that the combined effects of oxidative stress and increased inflammatory mediators served to decrease insulin expression and interfere with insulin biosynthesis in diabetes state [31]. The augmented reactive oxygen or nitrogen species (ROS/RNS) generation may play an important role in the diminished capability of beta cells. Interestingly, we noted that fenofibrate did not modulate circulating SOD concentration in obese MSG rats. Conversely, plasma MDA and NO levels (shown in Figure 1F and 1G) were dramatically increased and MDA level in pancreatic mitochondrion (shown in Figure 4A) of fenofibrate treatment group was augmented. These results indicate that fenofibrate promotes systemic inflammation and oxidative stress.

Furthermore, our results showed that total ATPase and Na^+^/K^+^-ATPase were decreased in pancreatic mitochondrion in fenofibrate treatment group (shown in Figure 4B and 4C), suggesting that the aggravation of lipid peroxidation may interfere with mitochondrial ion transport. Uncoupling-protein 2 (UCP2) is a mitochondrial protein located on the inner mitochondrial membrane. In pancreatic beta cells, UCP2 has been proposed as a negative regulator of glucose-stimulated insulin secretion [32]. We observed increased *UCP2 *mRNA level in the pancreas of fenofibrate treated obese MSG rats, thus we speculate that reduced insulin secretion after fenofibrate treatment may be induced by increased formation of oxidative products in the pancreas.

It is well known that high-fat diets or obesity result in the activation of NF- kappaB, leading to over-expression of its target genes such as *IL-6, iNOS, TNF alpha *[33,34]. The induction of iNOS and excess production of NO have been recognized as a major contributor to beta cell injury [35,36]. Our studies showed that plasma NO level was elevated and iNOS immunostaining was stronger in islet cells in fenofibrate treatment group. These observations indicated that iNOS-derived NO might serve as a physiological negative feedback inhibitor of acute glucose-stimulated insulin release in fenofibrate treated obese MSG rats [37,38]. Furthermore, we found that fenofibrate increased NF- kappaB, TNF alpha and iNOS expression both at mRNA and protein levels in the pancreas, but had no obvious effects on *PPAR alpha *expression. Collectively, our data strongly suggested that upregulation of NF- kappaB and iNOS in beta cells might contribute to the abnormal insulin secretion in fenofibrate treated obese MSG rats.

Our observation is a preliminary study to evaluate the effects of fenofibrate on beta cell function in animal models. Even though it may have some extent difference to the clinical patients, these findings could provide new insight into the role of PPAR alpha agonists in the regulation of beta cells function and call attention to the long term use of PPAR alpha agonists in the therapy of pre-diabetes and Type 2 diabetes patients.

## Conclusions

In summary, fenofibrate impaired *in vivo *insulin secretion stimulated by high glucose stimulation in obese MSG rats. A more pronounced effect on the first phase of insulin secretion suggests that ATP-dependent glucose sensing is impaired, which may be due to increased MDA level and decreased total ATPase activity in pancreatic mitochondrion. Up-regulated inflammatory mediators such as NF- kappaB and iNOS might contribute to the fenofibrate induced impairments of islet insulin secretion.

## Competing interests

The authors declare that they have no competing interests.

## Authors' contributions

SNL carried out studies including Real-time PCR, Western Blot and immunofluorescence assays and all the data analyses. SNL and QL performed the clamp test. LYL and SJS participated in the animal in vivo experimental test and biochemical analysis. YH participated in the primers sequence alignment. SNL and ZFS wrote the paper. ZFS designed the study and in coordination with all others drafted the manuscript. All authors read and approved the final version of the manuscript.
